# Novel Phage-Derived Depolymerase with Activity against *Proteus mirabilis* Biofilms

**DOI:** 10.3390/microorganisms9102172

**Published:** 2021-10-19

**Authors:** Cormac J. Rice, Stephen A. Kelly, Seamus C. O’Brien, Erinn M. Melaugh, Jan C. B. Ganacias, Zheng Hua Chai, Brendan F. Gilmore, Timofey Skvortsov

**Affiliations:** School of Pharmacy, Queen’s University Belfast, Belfast BT9 7BL, UK; crice41@qub.ac.uk (C.J.R.); Stephen.Kelly@qub.ac.uk (S.A.K.); Seamus.Obrien@qub.ac.uk (S.C.O.); Emelaugh01@qub.ac.uk (E.M.M.); Jganacias01@qub.ac.uk (J.C.B.G.); zchai01@qub.ac.uk (Z.H.C.); b.gilmore@qub.ac.uk (B.F.G.)

**Keywords:** *Proteus*, bacteriophage, urinary tract infections (UTIs), depolymerases, antibiotic resistance, pectate lyase, biofilms

## Abstract

The adherence of *Proteus mirabilis* to the surface of urinary catheters leads to colonization and eventual blockage of the catheter lumen by unique crystalline biofilms produced by these opportunistic pathogens, making *P. mirabilis* one of the leading causes of catheter-associated urinary tract infections. The *Proteus* biofilms reduce efficiency of antibiotic-based treatment, which in turn increases the risk of antibiotic resistance development. Bacteriophages and their enzymes have recently become investigated as alternative treatment options. In this study, a novel *Proteus* bacteriophage (vB_PmiS_PM-CJR) was isolated from an environmental sample and fully characterized. The phage displayed depolymerase activity and the subsequent genome analysis revealed the presence of a pectate lyase domain in its tail spike protein. The protein was heterologously expressed and purified; the ability of the purified tail spike to degrade *Proteus* biofilms was tested. We showed that the application of the tail spike protein was able to reduce the adherence of bacterial biofilm to plastic pegs in a MBEC (minimum biofilm eradication concentration) assay and improve the survival of *Galleria mellonella* larvae infected with *Proteus mirabilis*. Our study is the first to successfully isolate and characterize a biofilm depolymerase from a *Proteus* phage, demonstrating the potential of this group of enzymes in treatment of *Proteus* infections.

## 1. Introduction

*Proteus mirabilis* is a Gram-negative, opportunistically pathogenic bacterium which is widespread in natural and built environments and commonly present in gastrointestinal tracts of healthy humans. One of the distinguishing features of *P. mirabilis* is its swarming motility, resulting in formation of characteristic “bull’s-eye” patterns when grown on solid media. This is the result of sequential rounds of formation of elongated swarming cells, capable of flagella-assisted migration across surfaces, followed by deconsolidation into rod-shaped swimmer cells. Upon coming into contact with a surface and initial attachment, presumably facilitated by multiple types of fimbriae produced by *P. mirabilis* cells, the microorganisms undergo a drastic morphological change, elongating up to 50 times, and start secreting polysaccharides enabling attachment and colonization of surfaces in the form of biofilms. The readiness with which the bacterium forms biofilms on a variety of abiotic surfaces in combination with its ability to rapidly spread by swarming makes it a clinically important pathogen, capable of colonizing a variety of indwelling medical devices, including urinary catheters. It has been estimated that *P. mirabilis* causes between 1 and 10% of all urinary tract infections (UTIs) and is responsible for up to 44% of all catheter-associated urinary tract infections (CAUTIs) in the US [1]. One of the factors contributing to efficient urinary catheter colonization by *P. mirabilis* and resulting in severe UTIs is the production of a nickel containing metalloenzyme urease. Urease facilitates the hydrolysis of urea in urine, resulting in the formation of apatite and/or struvite crystals [2]. These crystals become embedded into the polymeric matrix of *Proteus* biofilms where they accumulate and grow, thus being a major factor in the blockage of urinary catheters. The obstructions created by crystalline biofilms prevent the free flow of urine and promote the ascending spread of infection, which ultimately leads to bacteriuria, pyelonephritis, bacteremia, and, in some cases, urosepsis and death. The socio-economic impact of CAUTIs, including those caused by *Proteus*, is significant, costing the National Health Service (NHS) between GBP 1.5 and 2.25 billion each year. Thus, a novel means of preventing and controlling crystalline biofilm formation is imperative [3].

In light of the ongoing antibiotics crisis and increasing prevalence of multidrug resistant strains among clinical isolates of *Proteus mirabilis* and related species, alternative approaches for control and management of CAUTIs are being investigated [4]. Among some of the most promising approaches for biofilm control is the use of bacteriophages. Bacteriophages are bacterial viruses that are the natural predators of bacteria and have been used in Eastern Europe and the former Soviet Union since before the discovery of antibiotics; after a long period of disuse, phage therapy has recently gained increased interest in Western medicine [5]. Phages act by binding to a receptor on the host cell surface before injecting their genetic material and replicating to produce progeny. The progeny eventually lyses the host cell and the cycle is repeated. The use of phages and their products as an alternative to traditional antibiotics has several advantages including high specificity and efficacy, low immunogenicity, and production costs [6,7]. Despite that, as both phage and their respective host are in a constant evolutionary arms race, the continuous isolation and characterization of novel bacteriophages is imperative for establishing an effective antimicrobial arsenal for treating a plethora of multidrug resistant infections [8,9].

**Figure 1:**
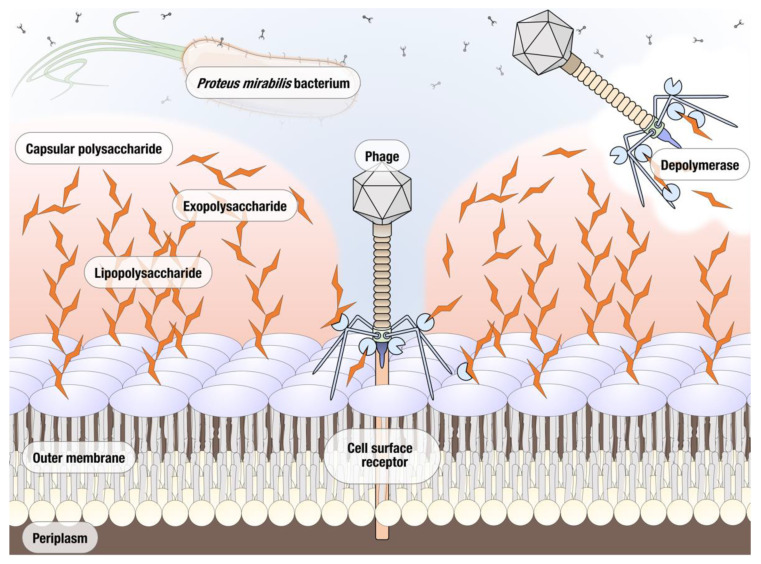
Potential polymeric targets for polysaccharide depolymerases of *Proteus* bacteriophages.

Phages are known to encode several enzymes of antimicrobial interest. Purification and characterization of phage derived antimicrobials allows for alternative phage-based therapeutics to be used in situations where the whole phage may not be as effective due to issues such as host specificity or resistance. In particular, the development of resistance appears to be happening at a significantly decreased rate when phage-derived enzymes are used instead of bacteriophages [10]. The most well studied of such enzymes are endolysins that degrade the peptidoglycan of the bacterial host’s cell wall; these enzymes have previously proven effective in controlling bacterial infections and a number are used commercially, e.g., phage enzyme preparations produced by Armata, Micreos, and Intralytix [11,12]. Another group of phage proteins with a significant therapeutic potential are polysaccharide depolymerases [13]. The majority of bacteriophage polysaccharide depolymerases are associated with the virion surface, most often encoded as a part of tail fibers and other structural proteins. Depolymerases are thought to act on either the capsular polysaccharide (CPS), the exopolysaccharides (EPS), or the lipopolysaccharides (LPS) of their host bacterium, cleaving these polymeric substances produced by the host cell and exposing the cell surface receptors necessary for binding, thus facilitating the phage infection (Figure 1). Due to the important role played by CPS, EPS, and LPS in the formation of biofilms, depolymerase enzymes are now being investigated in terms of preventing and treating biofilm related infections. The main advantage of phage depolymerases is their mode of action. Being non-lytic enzymes, depolymerases act as antivirulence agents, decreasing the severity of infection and helping the immune system of the host to clear the infection. Despite these attractive qualities, the research into therapeutic use of phage depolymerases so far has been mostly focused on a few bacterial species for which the critical importance of capsular polysaccharides as virulence factors has been well established, particularly in *Klebsiella pneumoniae*, *Acinetobacter baumannii*, and *Escherichia coli* [14,15,16]. As a result of their ability to prevent the formation and degrade established biofilms, depolymerase enzymes are now being explored as potential antimicrobials for the treatment of biofilm related infections. In this study, we isolated and characterized a novel lytic *Proteus mirabilis* phage vB_PmiS_PM-CJR capable of biofilm matrix degradation, identified a putative pectate lyase domain in its tail spike, and produced it as a recombinant protein. The ability of the recombinant depolymerase to reduce the formation of *Proteus mirabilis* biofilms in vitro and improve survival in *Galleria mellonella* infection (the greater wax moth larvae model often used to study host–pathogen interactions) was demonstrated experimentally. To the best of our knowledge, this is the first publication describing a functional depolymerase from a *Proteus* phage.

## 2. Materials and Methods

### 2.1. Bacterial Strains and Cultivation Media Used in the Study

*Proteus mirabilis* strain BB2000 [17] was used for isolation and propagation of bacteriophages. To determine the host range of isolated phages, 17 different clinically isolated and reference strains of *Proteus mirabilis* were used, in addition to *Proteus vulgaris* UM266 and *Proteus penneri* NCTC 12737 (see the Results section). All bacterial cultures were grown in LB broth (LBB) (Invitrogen, Paisley, UK).

### 2.2. Enrichment Cultures for Isolation of Proteus Bacteriophages

Mud samples collected from Shaw’s Bridge parkland region in Northern Ireland, UK were used for isolation of *Proteus* bacteriophages. For this, 1 mL of overnight cultures of several *Proteus* strains was inoculated with 0.5 g of the mud sample in addition to 10 mL of 10× strength LB growth media (Invitrogen, Paisley, UK) and 35 mL of autoclaved ultrapure water. The mixture was incubated for 3–5 days at 37 °C, 100 rpm to allow for the amplification of the phages. After the set period of time, the enrichment cultures were centrifuged at 4000× *g* for 5–10 min and the supernatant filtered through a Millex-GS 0.22 µm syringe filter (Millipore, Burlington, MA, USA). Spot tests of the enriched filtrates were performed against different strains of *Proteus* using the spot test overlay technique [18]. Briefly, 250 µL of bacterial overnight culture was inoculated into 5–6 mL of top agar (Invitrogen, Paisley, UK) (0.75% agar plus the other LB components) and quickly swirled before being poured on top of LB agar (Invitrogen, Paisley, UK) plates and left to set for 30 min. After the setting period, 10 µL of enriched 0.22 µm filtrate was spotted onto the plates and allowed to dry before being incubated at 37 °C overnight. The next day, phage spots were visualized, and their morphology and diameter recorded.

### 2.3. Plaque Assays and Isolation of Phage vB_PmiS_PM-CJR

The clear zone produced on *P. mirabilis* BB2000 lawn was picked and propagated on the respective host strain. Briefly, using a 10 µL sterile pipette tip, the point of the tip was touched against the center of the phage spot ensuring to avoid contact with the surrounding bacteria. The tip was then placed into 90 µL of sterile SM (phage) buffer [19] and consequently serially diluted with SM buffer 1 in 10 until a final dilution of 10^−8^ was achieved. To propagate phages, a plaque assay was performed, in which 10 µL of phage at different concentrations was inoculated with 250 µL of an overnight culture of the respective bacterial culture. The mixture was shaken gently by swirling before being left for 10 min to allow for phage adsorption to the bacterial cells. After the adsorption period, a top agar plaque assay was performed.

### 2.4. One-Step Growth Curve Assay

The one-step growth curve was performed as described elsewhere with small adjustments [20]. Briefly, a *P. mirabilis* BB2000 culture was incubated to early exponential phase and the bacterial culture adjusted to 1 × 10^8^ colony forming units (CFU)/mL. Then, 1 mL of the bacterial cell culture was centrifuged at 6000× *g* for 10 min and the pellet resuspended in 0.1 mL of 1 × 10^6^ plaque forming units (PFU)/mL of phage vB_PmiS_PM-CJR (MOI 0.001). The mixture was incubated at 37 °C for 15 min before being centrifuged twice at 5000× *g* for 3 min and washed with LBB to remove any unabsorbed phage from the mixture. The pellet was then resuspended in 9.9 mL of sterile LBB and the mixture incubated at 37 °C for 70 min at 250 rpm. Aliquots of the mixture were taken firstly every 5 min up to 20 min and then every 10 min thereafter. The aliquots taken were serially diluted and subject to a top agar plaque assay before the phage titer was determined and the burst size calculated. The burst size, expressed in plaque-forming units per infected cell (PFU/cell), was calculated as the average number of phage particles released between the end of the latent period and stabilization (plateauing) of the phage titer, and normalized by the titer of the bacterial culture.

### 2.5. Host Adsorption Assay

Host adsorption assay was carried out as described elsewhere [18,21]. Briefly, 950 µL of LB media and 60 µL of chloroform (Merck, Watford, UK) were added to 12 capped 13 × 100 mm sterile test tubes and chilled on ice for 10 min. A logarithmic phase culture of the respective host culture was diluted to give an OD_600_ of 0.2. To one flask was added 9 mL of the bacterial culture and to the other 9 mL sterile media. Both flasks were incubated at 48 °C for 5 min before 1 mL of phage suspension pre-warmed to 37 °C was added to each and a timer started. At 1 min intervals a 100 µL aliquot was taken and added to the chilled tubes. Finally, the tubes were vortexed vigorously and 100 µL samples taken and added to top agar with 250 µL host cells and a plaque assay performed. The plates were incubated at 37 °C until the appearance of plaques, after which the plaques were counted.

### 2.6. Host Range Determination of vB_PmiS_PM-CJR

The host range of the phage vB_PmiS_PM-CJR was determined via a spot assay against a range of *Proteus* spp. LB agar plates were overlaid with 5–6 mL of top agar containing 350 µL of the respective strain. Once dried, 10 µL of phage lysate was spotted on to the plate and allowed to dry before being placed in a 37 °C incubator for 18 h. After the incubation period, spots were observed on the bacterial lawns susceptible to vB_PmiS_PM-CJR infection.

### 2.7. Electron Microscopy

Transmission electron microscopy was performed by aseptically transferring 100 µL of high titer phage lysate into a 1.5 mL tube. The lysate was centrifuged at 4000× *g* for 22 min before being resuspended in 100 µL of sterile phage buffer. Using sterile EM forceps, a fresh EM grid (Ted Pella Inc., Redding, CA, USA) was placed on a clean KIM wipe with the dark shiny side facing up. Following this, 5 µL of phage lysate was added to the grid and allowed to sit for 8 min. The grid was rinsed at a 45° angle with 60 µL of ultrapure autoclaved water. Any excess water was allowed to drain off. Five microliters of 1% uranyl acetate were added to the grid and immediately removed after to prevent overstaining. The grid was allowed to dry overnight before being imaged on a JEOL JEM-1400 Plus Transmission Electron Microscope (JEOL Ltd., Tokyo, Japan).

### 2.8. Thermal Stability Test

To determine the thermal stability of phage vB_PmiS_PM-CJR, 500 µL of bacteriophage lysate with the initial titer of 1 × 10^10^ PFU/mL was incubated at the set temperatures (in the range from 40 to 100 °C) for an allocated period of time of 1 h. After the said period of time, the phage suspension was subject to a top agar overlay assay. The plates were incubated at 37 °C overnight and the plaques observed the next day were counted and quantified. Experiments were carried out in triplicate and the average number of plaques were used to determine the titer.

### 2.9. Extraction of Phage DNA

Phage DNA was extracted as follows. The lysate (500 µL) was treated with 1.25 µL of DNaseI (Sigma, Gillingham, UK) (20 mg/mL) and incubated at 4 °C overnight. The next day 1.25 µL of proteinase K (Sigma, Gillingham, UK) (20 mg/mL) was added to it along with 25 µL of 10% SDS and 20 µL of 0.5 M EDTA pH 8.0 (Merck, Watford, UK). The contents were mixed and incubated at 60 °C for 1 h. Once cooled, an equal volume of phenol:chloroform:isoamyl alcohol mixture (25:24:1 *v/v*) (Sigma, Gillingham, UK) was added and inverted several times before being centrifuged using an Eppendorf 5430 centrifuge (Eppendorf UK, Stevenage, UK) at 6000× *g* for 5 min at room temperature. Using a wide bore tip, the aqueous phase was transferred to a fresh 2 mL tube and the process repeated. The aqueous phase was once more transferred to a new tube where an equal volume of chloroform was added, after which the contents of the tube were again mixed by vortexing and centrifuged. The upper (aquatic) phase was transferred to a new tube and the previous step repeated once more. A 1/10 volume of 3M NaOAc (Thermo Fisher Scientific, Loughborough, UK)) (pH 7.5) was added along with 2.5 volumes of ice-cold molecular grade 95% ethanol (Sigma, Gillingham, UK) and mixed well, then incubated on ice for 30 min. The mixture was centrifuged at maximum speed for 20 min and the supernatant discarded, after which the DNA pellet was washed with an equal volume of 70% ethanol and reprecipitated by centrifugation for a further 2 min; this step was repeated one more time. The supernatant was discarded, and the remaining trace amounts of ethanol were allowed to evaporate before eluting the DNA in 30 µL of nuclease free water.

### 2.10. Phage vB_PmiS_PM-CJR Genome Seqeuncing, Assembly and Annotation

BGISEQ-500 WGS sequencing libraries were prepared and phage DNA was sequenced at BGI Hong Kong using the BGISEQ-500 sequencing platform (DNBSEQ technology; BGI, Hong Kong, China). Raw reads (150PE) were trimmed using Sickle v1.33 [22]. The processed reads were assembled using Unicycler v0.4.8, running in the conservative mode [23]. After that, PhageTerm v1.0.12 was used to determine the packaging type and predict phage termini [24]. The phage genome sequence was realigned in order to make the start of the sequence correspond with the predicted packaging site. Open reading frames were then predicted with Prodigal v3.6.3, after which the assembled phage genome was manually annotated in the Artemis genome browser tool (v18.1.0) [25,26], with the help of MultiPhATE (v2.1) and online versions of NCBI Blast (https://blast.ncbi.nlm.nih.gov/Blast.cgi; accessed on 21 July 2021), HHPred (https://toolkit.tuebingen.mpg.de/tools/hhpred; accessed on 21 July 2021), PhANNs (https://edwards.sdsu.edu/phanns; accessed on 21 July 2021), and HMMER (https://www.ebi.ac.uk/Tools/hmmer/; accessed on 21 July 2021) [27,28,29,30,31]. The putative tail spike protein of PmiS_PM-CJR was additionally analyzed with online versions of I-TASSER (https://zhanggroup.org/I-TASSER/; accessed on 1 July 2021), NCBI CDD (https://www.ncbi.nlm.nih.gov/Structure/cdd/wrpsb.cgi; accessed on 22 July 2021), and InterProScan (https://www.ebi.ac.uk/interpro/search/sequence/; accessed on 22 July 2021) [32,33,34]. Protein model visualization was performed with UCSF ChimeraX software (v1.2) [35]. Structural alignments were carried out using mTM-align (https://yanglab.nankai.edu.cn/mTM-align/; accessed on 22 July 2021) [36].

### 2.11. Whole-Genome Comparison and Phylogenetic Analysis

Easyfig v2.2.2 [37] was used to generate and visualize whole-genome comparisons of vB_PmiS_PM-CJR and most closely related phage genomes in the NCBI Genbank (identified as having at least 90% nucleotide sequence identity with at least 80% query coverage). Intergenomic similarities were calculated with the online version of VIRIDIC (http://rhea.icbm.uni-oldenburg.de/VIRIDIC/; accessed on 7 September 2021) using default parameters [38]. Amino acid sequences of the terminase large subunit (TerL) and major capsid protein (MCP) were used for phylogenetic characterization of vB_PmiS_PM-CJR. A BLASTp search against viral proteins available in NCBI RefSeq at the moment of writing was conducted and only proteins with sufficiently high similarity (e-value ≤ 1 × 10^−10^; percent identity ≥ 25%) to TerL and MCP of vB_PmiS_PM-CJR were retained for subsequent analyses. Multiple alignments of the amino acid sequences of TerL and MCP were generated in MAFFT v7.487 using L-INS-I settings, and TrimAl v1.4 was used for automated alignment trimming [39,40]. The inference of phylogenies was then carried out using IQ-Tree v2.1 [41], which was allowed to compute the optimal protein evolution model to be used. Visualization of the resulting tree files was subsequently performed with FigTree v 1.4.4 [42].

### 2.12. Phage vB_PmiS_PM-CJR Cloning and Expression of Tail Spike Protien

The gene construct containing the full tail spike protein gene was synthesized by Genscript (Leiden, the Netherlands). The synthesized gene was supplied in a pET-28a(+) plasmid vector (Novagen, Madison, WI, USA) cloned using BamHI and HindIII restriction sites. The plasmid construct was resolubilized to a concentration of 5 µg/mL in nuclease free water and 5 µL of this solution was used to transform *Escherichia coli* KRX cells (Promega, Madison, WI, USA, cat. no. L3002) using heat shock transformation. Briefly, 5 µL of the plasmid was added to 50 µL of KRX competent cells and left on ice for 30 min. An alternative pET-28a(+) vector with a different size gene and 5 µL of nuclease free water were used as positive and negative controls, respectively. The tubes were placed in a water bath at 42 °C for 1 min before returning to ice for 2 min. After that, 300 µL of SOC media (Thermo Fisher Scientific, Loughborough, UK) was added and the culture incubated at 37 °C for 1 h, followed by overnight incubation at 37 °C on pre-warmed LB agar plates containing 50 μg/mL kanamycin (Merck, Watford, UK).

### 2.13. Tail Spike Protein Expression and Purification

A single colony from transformation plates was used to inoculate LBB containing kanamycin (50 µg/mL) (Merck, Watford, UK) and 0.4% glucose (Merck, Watford, UK), and this was allowed to incubate at 37 °C overnight. Overnight starter cultures were diluted 1:100 into fresh LBB containing kanamycin (50 µg/mL) and grown at 37 °C at 200 rpm until an optical density (OD_600_) of 0.4 was achieved. The culture was then incubated at 25 °C until the OD_600_ reached 0.5, at which point 1 µL of 20% rhamnose (Merck, Watford, UK) was added along with IPTG (Merck, Watford, UK) to a final concentration of 1 mM. Cultures were allowed to incubate overnight at 25 °C before harvesting the cells via centrifugation at 10,000× *g* for 5 min. Cells were lysed via sonication on ice in sterile 1× Dulbecco’s phosphate-buffered saline (PBS) (BR0014, Oxoid, Basingstoke, UK).

Clarified by centrifugation (5000 rpm for 15 min) cell-free extract was purified by immobilized metal affinity chromatography (IMAC) using the N-terminal 6 × His-tag encoded by the pET-28a(+) vector. An ÄKTA Prime Plus Liquid Chromatography System (GE Healthcare, Chicago, IL, USA) was used to load the clarate on a HisTrap HP 1 mL column (both GE Healthcare Life Sciences, Little Chalfont, UK) at a rate of 1 mL/min. This was washed using buffer containing 50 mM NaH_2_PO_4_, 300 mM NaCl, and 20 mM imidazole, made up to 1 L with dH_2_O and adjusted to pH 8.0 with NaOH. Purified protein was eluted in 1 mL fractions using buffer containing 50 mM NaH_2_PO_4_, 300 mM NaCl, and 250 mM imidazole made up to 1 L with dH_2_O and adjusted to pH 8.0 with NaOH.

### 2.14. SDS-PAGE

Five microliters of 5× SDS-PAGE denaturing Laemmli buffer (20% 1.5 M Tris-HCl pH 6.8, 50% glycerol, 25% β-mercaptoethanol, 10% SDS (*w/v*), and 5% of 1% bromophenol blue) was added to 20 µL of respective sample and heat treated at 95 °C for 10 min. Ten microliters of each denatured sample was loaded into an Invitrogen NuPAGE 4–12% Bis-Tris gel (1.5 × 10 mm well; Invitrogen, Carlsbad, CA, USA) along with 5 µL of SeeBlue Plus2 Pre-stained Protein Standard (Invitrogen, Carlsbad, CA, USA) and run at 200 V for approximately 1 h, using Novex 1× MES SDS (Invitrogen, Carlsbad, CA, USA) as running buffer. The gel was stained using Coomassie blue staining solution (2% Coomassie blue R-250 (*w/v*), 42.5% water, 50% ethanol and 7.5% acetic acid). The gel was then placed in de-stain solution (87.5% ddH_2_O, 5% methanol and 7.5% acetic acid) and washed three times with water. The gel was imaged using G:BOX Chemi XRQ imager (Syngene, Cambridge, UK).

### 2.15. Reverse Phase High Performance Liquid Chromatography (RP-HPLC) for Tail Spike Purity Analysis

The purity of the recombinant tail spike protein was determined using an Agilent 1260 Infinity (Agilent Technologies, Santa Clara, CA, USA) RP-HPLC system. Ten microliters of the purified tail spike (891 ng/µL) was loaded onto a Kinetex C18 column (50 × 4.6 mm, 5 µm, 100 Å; Phenomenex, Macclesfield, UK) and analyzed at room temperature. Two mobile phases were used: 0.1% trifluoroacetic acid (TFA) in water (mobile phase A) and 0.1% TFA in acetonitrile (mobile phase B). The mobile phase gradient used was 5% to 95% B in 30 min, with a flow rate of 1 mL/min. Protein elution was continuously monitored by measuring the absorbance at 254 nm wavelength.

### 2.16. Assessing the Activity of vB_PmiS_PM-CJR’s Recombinant Tail Spike Protein

To assess the ability of the protein to degrade the polysaccharide matrix produced by *Proteus*, a dilution series of the purified tail spike protein prepared in sterile 1× PBS buffer was used in a spot test and compared to serial dilutions of vB_PmiS_PM-CJR, also diluted in sterile 1× PBS. A top agar spot test on a lawn of *P. mirabilis* BB2000 was performed before incubating the plates overnight at 37 °C. Spot zones were observed the next day and compared.

### 2.17. P. mirabilis MBEC Peg Adsorption Assay

The MBEC (Innovotech Inc, Edmonton, AB, Canada) adherence assay was performed by adjusting the OD_600_ of an overnight culture of *P. mirabilis* BB2000 to 0.1 in sterile LBB media. The optical density was adjusted using LB broth with tail spike protein added to a final concentration of 100 µg/mL. After that, 150 µL of culture was then added to the rows of the MBEC plate. The MBEC lid with pegs protruding from the surface was added to the bacterial culture wells supplemented with tail spike protein and placed into a plastic container with a damp lint free cloth. The container with the MBEC plate was incubated at 37 °C for 4 h before colony counts were performed. Control wells of LBB and a vehicle control (50 mM Tris-HCl pH 8.0 diluted into sterile LBB) were added to the MBEC plate. Phage vB_PmiS_PM-CJR solution was used for comparison (final titer—1 × 10^6^ PFU/mL). The collected data was analyzed using Prism 7 (GraphPad Software, San Diego, CA, USA). One-way ANOVA with Tukey’s post-hoc test was used for comparison of treatment groups.

### 2.18. Galleria mellonella Survivability Study

Fresh *Galleria mellonella* (greater wax moth) larvae were used for this study. Larvae were injected with 20 µL of an overnight culture *P. mirabilis* BB2000 at a range of different titers diluted down using sterile 1× PBS. Using sterile 29-gauge insulin needles, 20 µL of the bacteria was injected into the lower left anal end proleg and the larvae placed in a sterile Petri dish. For treatment conditions, 20 µL of the treatment solution to be used was immediately injected into the lower right proleg at the anal end of the larva after injection of the bacteria. Treatment of both phage and recombinant tail spike protein were used. Phage vB_PmiS_PM-CJR was used at a titer of 1 × 10^7^ PFU/mL. The tail spike protein was administered at a concentration of 100 µg/mL. Controls of 1× PBS were used to ensure the correct injecting technique was used. Larvae were incubated at 37 °C and survivability recorded at 24 and 48 h time points. Experiments were carried out using 10 replicates for each condition and repeated three times. Survivability was determined as the percentage (%) of *G. mellonella* alive and motile after the defined incubation periods.

## 3. Results

### 3.1. Biological Characteristics

#### 3.1.1. Phage Isolation and Purification

**Figure 2:**
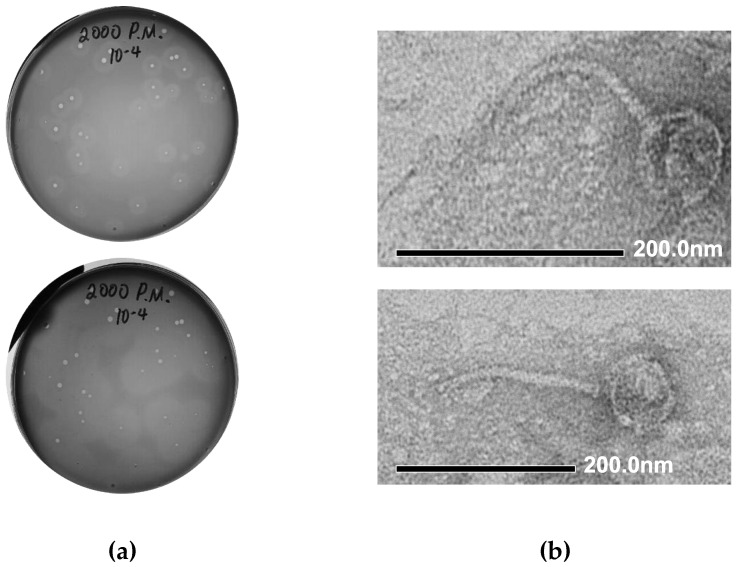
Morphological characteristics of vB_PmiS_PM-CJR. (**a**) Plaque morphology. The presence of semi-turbid haloes surrounding the plaques proper is visible after 24 h (top). Expansion of haloes after 48 h (bottom). (**b**) Transmission electron microscopy of phage particles. Phage particles were stained with 1% uranyl acetate and visualized at 40,000× (top) and 30,000× (bottom) magnification.

We performed the isolation of *Proteus* bacteriophages from a number of environmental samples collected in Northern Ireland (mud samples collected from the vicinity of the Shaw’s Bridge on River Lagan), using *Proteus mirabilis* BB2000 as a host strain. Initial identification of the presence of phage activity was conducted using spot assays. The plaque assay conducted with the material from one of the phage-positive samples led to the formation of medium-sized (1–3 mm) clear plaques on a lawn of *P. mirabilis* BB2000, surrounded by semi-transparent haloes (Figure 2a, top). The halo region around each plaque is indicative of phage encoded depolymerase enzyme activity and the expansion of the halo region after 48 h, as seen in Figure 2a, bottom, is also characteristic of phage depolymerases. The material from one of the plaques was used for further phage purification by three sequential plaque assays. The resulting phage stock suspension was prepared in SM buffer and stored at +4 °C.

#### 3.1.2. Electron Microscopy

The morphology of phage particles was examined by transmission electron microscopy (TEM). Based on the appearance, the phage could be described as a siphovirus, with viral particles having the icosahedral capsid (76 nm in diameter) and a long, flexible tail, approximately 180 nm long (Figure 2b). Using the recommended phage naming guidelines [43], the newly isolated phage was named vB_PmiS_PM-CJR.

#### 3.1.3. Infection Kinetics

**Figure 3:**
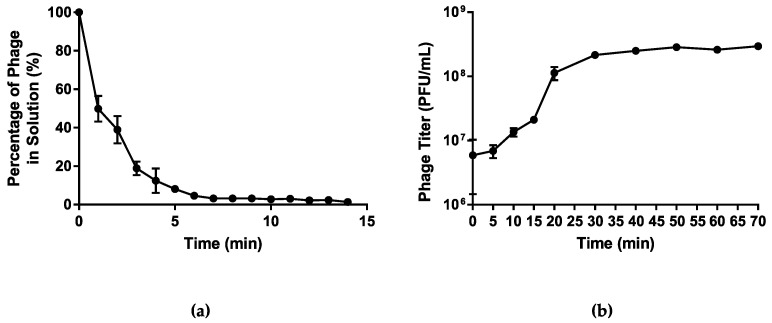
Growth characteristics of vB_PmiS_PM-CJR. (**a**) The host adsorption assay. The percentage of the phage that did not adsorb to the host cells is shown on the y axis. (**b**) One-step growth curve. Phage titer at different times is shown on the y axis, time point 0 corresponds to the moment of the first plating (26 min post-infection). Mean values ± SD (standard deviation) of three independent experiments are plotted.

Host adsorption assay was used to assess the rate of attachment of phage vB_PmiS_PM-CJR to its host cell surface receptors. The analysis of the data obtained showed that over 90% of the phage particles adsorbed to the host cells within first 5 min after the phage addition (Figure 3a). The one-step growth curve analysis was used to characterize the life cycle of vB_PmiS_PM-CJR and assess its growth kinetics, including the latent period and burst size (Figure 3b). The latent period of phage vB_PmiS_PM-CJR was estimated to be approximately 10 min, which can be considered average in comparison to other *Proteus* phages [44,45,46], although direct comparison is difficult due to substantial methodological differences between studies. The growth cycle appears to be complete in approximately 30–35 min as the phage titer begins to plateau after this time period. The burst size of the phage was determined to be approximately 30 PFU/cell.

#### 3.1.4. Phage Host Range

The host range of the phage was determined against 17 different clinical and reference isolates of *P. mirabilis*, one strain of *P. vulgaris*, and one strain of *P. penneri*. Spot tests were performed via top agar overlay technique revealing that the phage was able to infect nine of the 14 *P. mirabilis* strains tested and was able to infect both the *P. vulgaris* and the *P. penneri* strains (Table 1).

#### 3.1.5. Thermal Stability

**Figure 4:**
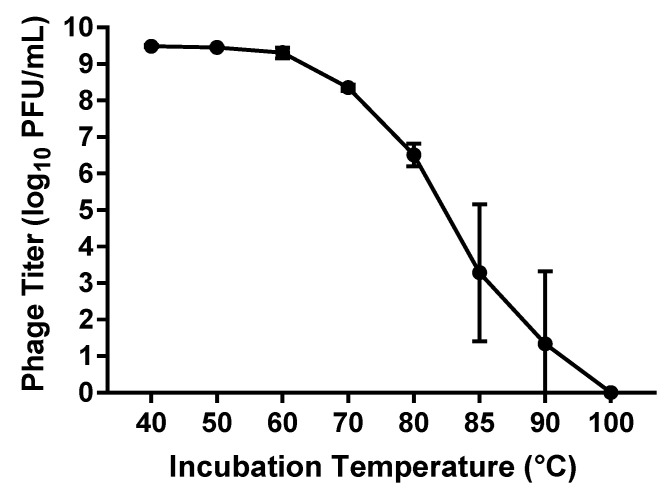
Thermal stability assay results. The titer (log_10_ PFU (plaque forming units)/mL) of phage remaining after incubation at temperatures from 40 to 100 °C for 1 h is shown. Mean values ± SD of three independent experiments are plotted.

The thermal stability of phage vB_PmiS_PM-CJR was assessed by exposing a 500 µL sample of phage (1 × 10^10^ PFU/mL) to a range of temperatures from 40 to 100 °C for the duration of 1 h, after which the titer of the phage was measured using double agar plaque assay. The data seen in Figure 4 shows that the virus is relatively thermally stable as the titer remains reduced less than 1 log_10_ until 70 °C, with temperatures below 60 °C having virtually no effect on the viability of the phage. The presence of viable phage particles was still detected even after incubations at temperatures of 85 and 90 °C for 1 h.

### 3.2. Genome Analysis and Phylogenetic Comparison

#### 3.2.1. General Characterization of the vB_PmiS_PM-CJR Genome

The linear dsDNA genome of the phage vB_PmiS_PM-CJR was sequenced to describe its main genomic features and determine the localization of the gene encoding polysaccharide depolymerase. A single contiguous sequence of 54,169 bp was assembled from 8,409,016 reads with more than 150× coverage. An analysis of the assembled genomic sequence conducted with PhageTerm suggested that vB_PmiS_PM-CJR utilizes a headful packaging mechanism (P1 type); the DNA sequence was rearranged to align the start of the sequence with the predicted packaging site. The GC content of the phage was found to be 36.3%, which is slightly lower than that of the isolation host strain *Proteus mirabilis* BB2000, whose GC content is 38.6% [17]. This is in line with the described tendency of intracellular parasites to have lower GC content than their hosts [53].

**Figure 5:**
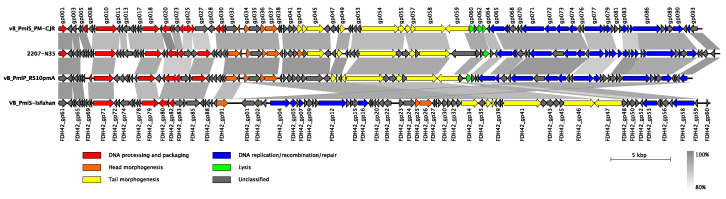
Genomic organization and whole-genome comparison of vB_PmiS_PM-CJR and closely related phages. Areas of substantial similarity between two genomes are shown as trapezia connecting two genome regions colored according to average nucleotide identity levels of these regions (as calculated by BLASTn). Arrows on the genome map represent identified genes and demonstrate the direction of their transcription. They are colored to reflect common functions of the encoded products. Putative genes with unidentified function are shown in grey color. Original functional annotations were used for each of the genomes.

In terms of the genetic composition, ninety-three putative open reading frames (ORFs) were predicted in the vB_PmiS_PM-CJR genome, with 41 ORFs on the positive strand and 52 ORFs on the negative one (Figure 5). The coding percentage was estimated to be 94.4%, and no tRNA genes or other non-coding RNA genes were identified. Only 47 of 93 ORFs (50.5%) could be functionally annotated; the remaining genes were designated as encoding hypothetical proteins. Among the annotated genes, genes of DNA processing and packaging machinery, structural and lytic proteins, and the genes encoding various components of replication, recombination, and repair molecular mechanisms were identified, all forming distinct functional modules. No genes coding for proteins with significant similarity to those associated with temperate lifestyle (e.g., integrases, repressor proteins) were identified, suggesting that the phage vB_PmiS_PM-CJR is lytic. Similarly, no genes encoding auxiliary metabolic genes, virulence factors, toxins, or antibiotic resistance genes were found among the annotated genes. An annotated genome sequence of the phage vB_PmiS_PM-CJR was deposited in NCBI GenBank under the accession number MZ643249.

#### 3.2.2. Whole Genome Comparison and Phylogenetic Analysis

Based on the overall nucleotide identity to the vB_PmiS_PM-CJR genomic sequence, three closely related bacteriophages were identified in NCBI Genbank. These bacteriophages were annotated as *Proteus* phages 2207-N35 (MN840487.1), vB_PmiP_RS10pmA (MG575420.1), and VB_PmiS-Isfahan (NC_041925.1), all being classified as members of the *Gorganvirus* genus in the *Siphoviridae* family. The results of comparison of genome structure and organization of all four closely related phages are presented in Figure 5. Although all genomes are quite similar in terms of length, overall sequence identity, and gene number, several differences can be seen in the illustration. The most noticeable is a large inversion present in the genome of VB_PmiS-Isfahan compared to the other three phages. A number of regions of low sequence identity are also present, the majority of which are concentrated in genes encoding proteins with unknown function, mainly co-localized with early genes responsible for hijacking of the transcriptional and translational machinery of the host cell. Another noticeable difference is present in the 3′ end of the tail spike protein (gp059 in vB_PmiS_PM-CJR) responsible for host recognition and infection.

A comparison conducted using VIRIDIC allowed us to tentatively place vB_PmiS_PM-CJR in the same *Gorganvirus* genus as three other analyzed phages (all phages had intergenomic similarity in the range 82–85%). To further establish the phylogenetic relationship of vB_PmiS_PM-CJR with other tailed phages, two highly conserved genes, major capsid protein (MCP; gp037) and the large terminase subunit (TerL; gp010), were used to generate phylogenetic trees (Figure S1). The comparison of the resulting trees suggests that vB_PmiS_PM-CJR is most closely related to three other *Gorganviruses* of *Proteus* and to *Salmonella* phages 9NA, SP069 (*Nonanviruses*) and *Sashavirus* vB_SenS_Sasha. These results are similar to those previously reported for VB-PmiS-Isfahan [54].

#### 3.2.3. Putative Polysaccharide Depolymerase of vB_PmiS_PM-CJR

Phage structural proteins, such as tail fibers and tail spikes, often contain domains with polysaccharide depolymerase activities. The tail spike protein (gp059) was analyzed with NCBI CDD and InterProScanA, revealing the presence of a pectin lyase-like fold (InterProScan IPR011050; residues 173–434) and some similarity to pectate_lyase_3 domain (pfam12708; residues 170–221), whereas the N-terminal region (residues 1–128) had a high degree of conservation between vB_PmiS_PM-CJR and three other *Gorganviruses*. A comparison of amino acid sequences of tail spikes of all four phages can be found in Figure S2a.

Protein modeling of the tail spike was performed in I-TASSER (Figure S2b). The resulting model had a C-score of –1.06 and the estimated TM-score was 0.58 ± 0.14, signifying acceptable modeling confidence and indicating the correct topology of the generated model. The tail spike protein model was used for a subsequent structural comparison to the PDB (v 2019-08-04) with mTM-align, which revealed high similarity of the model to tail spike proteins 4 (TSP4DN; ORF213; TM-score > 0.91) and 2 (TSP2DN; ORF211; TM-score > 0.59) of the bacteriophage CBA120 [55].

### 3.3. Phage Depolymerase Characterization

#### 3.3.1. Tail Spike Protein Expression, Purification and Confirmation of Enzymatic Activity

**Figure 6:**
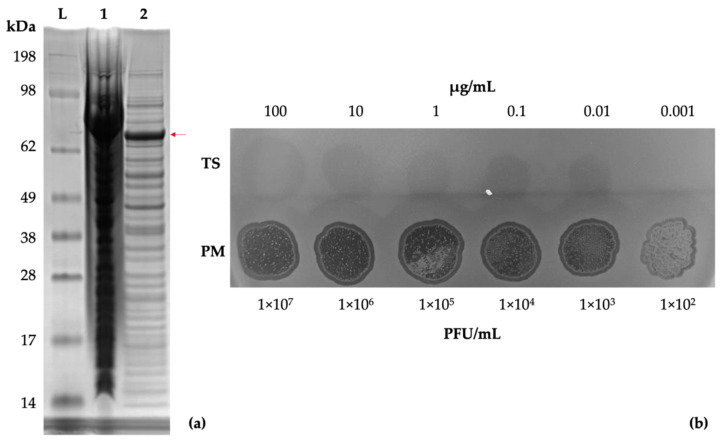
Tail spike expression and confirmation of activity. (**a**) SDS-PAGE gel of *E. coli* KRX cells expressing the recombinant tail spike protein. Lanes: SeeBlue Plus2 reference ladder (L), total protein (1), soluble protein fraction (2). A band of approximately 72 kDa, corresponding to a monomer of the tail spike protein, is visible (indicated with a red arrow). (**b**) Spot testing (10 µL) of the purified tail spike protein and phage suspension on a lawn of *P. mirabilis* BB2000.

Genomic analysis of vB_PmiS_PM-CJR suggested the presence of a putative pectin lyase in its tail spike protein. The gp059 gene construct was synthesized by Genscript and the resultant plasmid was expressed in *E. coli*. A band of the expected size (72 kDa) was visible on an SDS-PAGE (Figure 6a). The recombinant protein was purified for subsequent experiments using the ÄKTA Prime Plus chromatography system (Figure S3).

A dilution series of the purified tail spike was prepared and tested by a spot test to assess its enzymatic activity, using vB_PmiS_PM-CJR for comparison (Figure 6b). The test confirmed that the protein retained its depolymerase activity in its isolated form.

#### 3.3.2. Antibiofilm MBEC Adherence Assay

**Figure 7:**
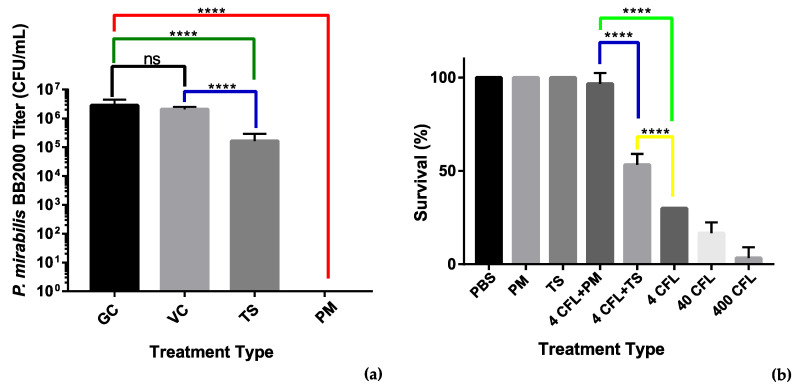
Analysis of antimicrobial activity of the phage vB_PmiS_PM-CJR and its tail spike. (**a**) MBEC (minimum biofilm eradication concentration) adherence assay. Treatments: GC—growth control, VC—vehicle control (50 mM Tris-HCl pH 8.0 in LBB), TS—tail spike dialyzed into vehicle, PM—phage vB_PmiS_PM-CJR in 1× PBS. (**b**) *Galleria mellonella* infection model. The number of surviving larvae 48 h post-treatment are shown. Treatments (all in 1× PBS): PBS—buffer only, PM—phage only, TS—tail spike only; 4, 40, and 400 CFL—*Proteus mirabilis* injections of 4, 40, and 400 CFU (colony forming units)/larva. Modifiers +PM and +TS are used to indicate administration of either 2 × 10^5^ vB_PmiS_PM-CJR phage particles or 2 µg of the purified tail spike protein per larva, respectively. Both experiments were conducted in triplicate, mean values ± SD are reported; ns—not significant, ****—*p* < 0.0001 (one-way ANOVA with Tukey’s post-hoc test).

We used an MBEC assay to assess the antibiofilm properties of the recombinant tail spike protein. An overnight culture of *P. mirabilis* BB2000 diluted to OD_600_ 0.1 was added to LB broth supplemented with the tail spike protein to a final concentration of 100 µg/mL. The adherence of the bacteria to MBEC pegs was assessed after a 4 h timepoint. Colony counts of the sonicated pegs revealed that the presence of the tail spike protein was able to account for a 10-fold reduction in bacteria that had adhered to the MBEC pegs. Statistical analysis revealed the difference in colony counts between the treated group and the untreated group was significant with *p* < 0.0001 (Figure 7a). The reduction observed may indicate that the tail spike protein holds antimicrobial potential against *P. mirabilis* and their associated infections.

#### 3.3.3. *Galleria mellonella* Survivability Study

To further assess the antimicrobial activity of the tail spike protein, a *G. mellonella* survivability study was designed in which the recombinant tail spike was injected (100 µg/mL) immediately after injection of different titers of *P. mirabilis*. The whole phage vB_PmiS_PM-CJR was also used in the study to assess its antimicrobial potential. Injection of the phage (1 × 10^7^ PFU/mL) at both bacterial concentrations significantly increased the survivability of the larvae (Figure 7b). The phage was also shown to be non-toxic to the larvae at the titers up to 1 × 10^11^ PFU/mL (data not shown). Injection of the tail spike protein was also found to be effective at improving *G. mellonella* survivability after 48 h when compared to the untreated controls. The whole phage allowed for 40% increased survival when compared to the tail spike treatment. Application of the tail spike facilitated an increased 20% survival when compared to the untreated control group.

## 4. Discussion

Biofilm formation is a characteristic property of many microorganisms causing medical device-associated infectious diseases. Among such infections, CAUTIs constitute a significant proportion and *Proteus mirabilis* is responsible for a substantial number of them, especially in patients with long-term catheterization and in case of complicated UTIs [1,56]. Due to its unique ability to form crystalline biofilms, established *P. mirabilis* infections are difficult to treat with conventional antibiotics [56]. In addition, the prevalence of antibiotic-resistant *Proteus* spp. strains is increasing, necessitating a more intensive search for alternative approaches [57]. The use of therapeutic bacteriophages and their enzymes for biofilm control is a promising research direction—a number of phage cocktails have been tested in clinical trials, including a recently finished one, in which bacteriophage therapy was found to be non-inferior to the standard-of-care antibiotic treatment of UTIs [58,59,60,61]. The research into the application of bacteriophages against *Proteus* has attracted a certain amount of attention (e.g., [45,62]), with a recent study reporting encouraging results of the use of phage-functionalized hydrogels for the prevention of catheter blockage [60].

In contrast to whole-phage preparations, isolated natural and genetically engineered enzymes of *Proteus* phages (endolysins, polysaccharide depolymerases) have not been intensively investigated as antibacterial agents, especially in comparison with other Gram-negative pathogens [63,64,65]. In a recent work, Alves et al. reported that the majority of *Proteus* phages they studied produced clear plaques surrounded by haloes, which is a well-known characteristic of phage depolymerase activity; despite that, the authors were unable to identify the ORFs encoding potential depolymerases in the genomes of the bacteriophages they sequenced [66]. As it was shown previously that other phages effective in controlling biofilm formation by *Proteus mirabilis* produce expanding haloes [67], we attempted to isolate the factor responsible for this activity from a novel halo-forming bacteriophage whose microbiological and genetic characteristics we describe in the present study.

The genome sequencing and comparative bioinformatics analysis revealed that the isolated bacteriophage is a new representative of a recently established genus of *Gorganviruses*, *Siphoviridae*, whose first member was described in the publication by Yazdi et al. [54]. The bioinformatics analysis of our phage vB_PmiS_PM-CJR was in agreement with the microscopy results, which confirmed its siphoviral morphology. Although the genome sequences of vB_PmiS_PM-CJR and three other *Gorganviruses* have substantial nucleotide identity, they together are relatively distant from other bacteriophage groups. Further analyses based on reconstruction of phylogenetic trees using MCP and TerL proteins have also demonstrated that vB_PmiS_PM-CJR is closely related to *Gorganviruses* and (to a less extent) to a number of other *Siphoviridae*, most notably genera *Noravirus* and *Sashavirus* of *Salmonella.* The phylogenetic relationships with more distantly related phages are less obvious from the trees, as their topologies and phages constituting them differ significantly.

The majority of phage polysaccharide depolymerases are found within or in close proximity to genes of tail fibers and tail spikes, and thus can be considered structural proteins [68]. Although polysaccharide depolymerases are structurally similar, central regions of the genes encoding them are highly variable and can significantly differ even between closely related phage species [69]. As a comparative genomic analysis showed the absence of sequence conservation in the gene of a tail spike protein of vB_PmiS_PM-CJR and the presence of a putative pectate lyase domain was predicted by InterProScan and NCBI CDD, we decided to investigate the tail spike in more detail to verify the presence of polysaccharide depolymerase in it.

The purified tail spike was tested using a lawn spot test alongside the vB_PmiS_PM-CJR. The formation of semi-turbid spots similar in appearance to haloes surrounding phage plaques confirmed the presence of polymer-degrading enzymatic activity. To further assess the activity of the depolymerase and its potential applicability as an antibiofilm agent, the enzyme was tested in an MBEC assay. The enzyme interfered with the attachment of *Proteus* to plastic pegs of the MBEC device, reducing the number of adsorbed bacteria by 90%, indicating the protein may hold potential for catheter treatment and prevention of *P. mirabilis* colonization of catheters. Finally, we showed that an injection of the tail spike protein reduces mortality of *G. mellonella* from *P. mirabilis*, with five of 10 larvae surviving after 48 h post-infection with 4 CFU/larva, compared to three of 10 in the control group on average. Due to the fact that the larval immune system of *G. mellonella* has a number of similarities to the innate immune response of humans and other mammals, it is widely used as a model to study host–pathogen interactions and immune responses [70]. This model has previously been applied to assess the efficiency of different antimicrobial agents, including bacteriophages and their enzymes [71,72]. As *Proteus mirabilis* is highly lethal to *G. mellonella* larvae (previously reported LD_50_ are in the range of 10–100 CFU/larva, which is in agreement with our findings) [73,74], our results demonstrate that the depolymerase has a noticeable antivirulence effect.

Polysaccharide elements appear to play a major role in the genesis of *Proteus* biofilms, forming a foundation layer [75]. Although we showed that the tail spike has an effect on *Proteus* cells, its exact target is currently unknown. Pectin/pectate lyases (EC 4.2.2.10 or 4.2.2.2) are among the most common depolymerases produced by phages [68] and are known to degrade negatively charged non-methylated, low-esterified substrates, by acting on the α-1,4 bonds between galacturonosyl residues [76]. The presence of a pectate lyase domain implies that the enzyme is capable of degrading polygalacturonic acid, a major component of bacterial polysaccharides. Galacturonic acid and its derivatives are frequently present in O-antigens of *Proteus* lipopolysaccharides (LPS), which may be the target of the tail spike activity [77]. Interestingly, structural modeling of the tail spike suggested that it is structurally similar to the hydrolysases TSP4 (PDB: 5w6h) encoded by ORF213 *E. coli* phage CBA120 and, to a lesser extent, to TSP2 from the same phage [55,78]. Nevertheless, although it may indicate that the tail spike of vB_PmiS_PM-CJR has similar activity, the low general accuracy of computational protein structure prediction of phage proteins and high level of structural conservation among phage depolymerases mean that the depolymerase target cannot be established reliably with this approach. Alternatively, the tail spike might be active against a capsular polysaccharide (CPS) produced by *Proteus* cells. A number of publications report the presence of a capsule and its involvement in the formation of crystalline biofilms by *Proteus mirabilis* [4,79,80,81,82,83,84], although not all strains of *Proteus* can synthesize it [85]; no capsule was detected following multiple capsule staining attempts of *P. mirabilis* BB2000 (data not shown). In *Proteus* strains that produce CPS, its structure appears to be identical to O-specific chains of their LPS [79,85,86]. Finally, a possibility of existing of another polymeric target (e.g., EPS of the glycocalyx) for the tail spike cannot be excluded.

Arguably, one of the main limitations to implementing phage depolymerases as antivirulence agents, whether alone or in combination with other antibacterials, is their narrow spectrum of activity. Although the host range of phage vB_PmiS_PM-CJR included a number of strains of *P. mirabilis*, in addition to *P. vulgaris* and *P. penneri* strains, the recombinant tail spike protein was limited to just its original vB_PmiS_PM-CJR isolation strain *P. mirabilis* BB2000 (Table 1), indicating that the tail spike protein is very specific to this particular host or that its binding to the components of the polymeric matrix was not followed by their enzymatic degradation. The presence of other receptor-binding proteins additional to gp059 in vB_PmiS_PM-CJR may also explain the wider host range of the phage compared to the depolymerase. Although the narrow activity spectrum of phages is considered beneficial for clinical applications as it limits the effect of the phage on non-pathogenic constituents of the microbiota, protein engineering of the tail spike can be employed to expand its host range [87,88,89].

Phage polysaccharide depolymerases are promising antibacterial agents and tools for studying bacterial polysaccharides and phage–bacteria co-evolution. We demonstrated that the isolated tail spike has potential to reduce colonization of polymer surfaces by interfering with the formation of *Proteus* biofilms and improves survival of infected *Galleria mellonella*. We are planning to continue our investigation to better describe the isolated depolymerase by identifying the target polysaccharide and conducting its additional biochemical characterization. It should be noted that a phage depolymerase with promising antivirulence properties was recently identified in a podovirus infecting *Providencia stuartii*, an emerging nosocomial pathogen [90]. As *Proteus, Providencia*, and *Morganella* are closely related, it would be interesting to further investigate the diversity and the spectrum of activity of depolymerases from phages of that group of bacteria and attempt to identify ones with a broader range of targets.

## 5. Conclusions

In this study we isolated a new *Proteus* bacteriophage and determined that its recombinant tail spike protein has polysaccharide depolymerase activity. To the best of our knowledge, this is the first polysaccharide depolymerase from a *Proteus* bacteriophage described in the literature. We experimentally demonstrated its antibacterial properties and believe that our results may contribute to development of novel approaches of treatment and prevention of CAUTIs caused by *Proteus*.

## Figures and Tables

**Table 1:** Host range determination of the purified phage vB_PmiS_PM-CJR and its recombinant tail spike protein.

Species	Source	Phage	Tail Spike	Reference
*P. mirabilis* BB2000	Reference strain	+++	+++	[17]
*P. mirabilis* ATCC 51286	Reference strain	+++	–	–
*P. mirabilis* ATCC 35508	Reference strain	++	–	–
*P. mirabilis* HI4320 *ure^−^*	Urease-negative mutant	–	–	[47]
*P. mirabilis* B2	Clinical isolate	+	–	[48]
*P. mirabilis* B4	Clinical isolate	++	–	[49]
*P. mirabilis* RB6	Clinical isolate	–	–	[50]
*P. mirabilis* RB6A	Clinical isolate	–	–	–
*P. mirabilis* RB6B	Clinical isolate	–	–	–
*P. mirabilis* RS1	Clinical isolate	–	–	[51]
*P. mirabilis* RS6	Clinical isolate	–	–	[52]
*P. mirabilis* RS17	Clinical isolate	–	–	[52]
*P. mirabilis* RS18	Clinical isolate	–	–	[52]
*P. mirabilis* RS28	Clinical isolate	+	–	[52]
*P. mirabilis* RS40	Clinical isolate	++	–	[52]
*P. mirabilis* RS47	Clinical isolate	++	–	[52]
*P. mirabilis* RS50a	Clinical isolate	+	–	[52]
*P. penneri* NCTC 12737	Reference strain	+	–	–
*P. vulgaris* UM266	Clinical isolate	+	–	–

The level of effect of phage and the tail spike on different bacterial strains was qualitatively assessed and expressed using the following convention: strong effect (+++), moderate effect (++), weak effect (+), no detectable effect (–).

## Data Availability

All data produced in this study is available in the main text of the publication or in the Supplementary Materials.

## Supplementary Materials

The following are available online at https://www.mdpi.com/article/10.3390/microorganisms9102172/s1. Figure S1: Phylogenetic trees based on amino acid sequences of terminase large subunit and major capsid protein of phage vB_PmiS_PM-CJR and related bacteriophages. Figure S2: Multiple sequence alignment of the tail spike protein sequences of vB_PmiS_PM-CJR and related *Gorganviruses* and a structural model of the vB_PmiS_PM-CJR tail spike generated with I-TASSER. Figure S3: Purified protein RP-HPLC analysis results.
