# Supplementary material for: Novel Phage-Derived Depolymerase with Activity against Proteus mirabilis Biofilms
Source: Microorganisms. 2021 Oct 19;9(10):2172. doi: 10.3390/microorganisms9102172 (PMC8539402; doi:10.3390/microorganisms9102172)
Supplement: Supplementary file 1 [file microorganisms-09-02172-s001.zip › microorganisms-1420239-supplementary.pdf]

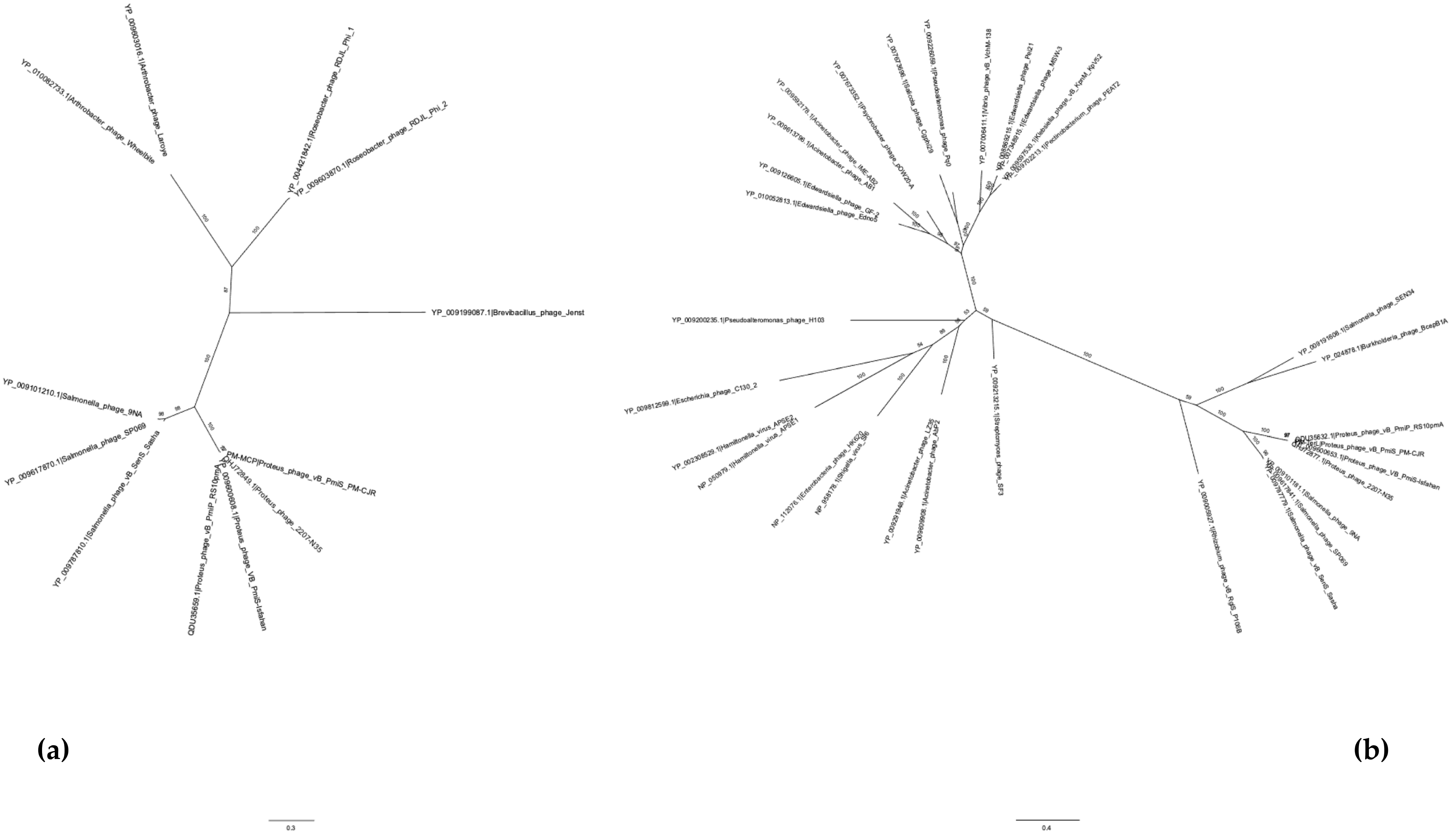

Figure S1. Phylogenetic trees based on amino acid sequences of conserved proteins of phage vB_PmiS_PM-CJR and related bacteriophages. (**a**) Phylogenetic analysis of terminase large subunits. (**b**) Phylogenetic analysis of major capsid proteins. Multiple sequence alignments were generated by MAFFT in L-INS-I mode and trimmed with TrimAl. Phylogenetic trees were reconstructed with IQ-TREE 2 and visualized with FigTree, with bootstrap support values reported over tree branches. As the analysis was used to understand phylogenetic relationships of phages rather than reconstruct their evolutionary history, no rooting was applied.

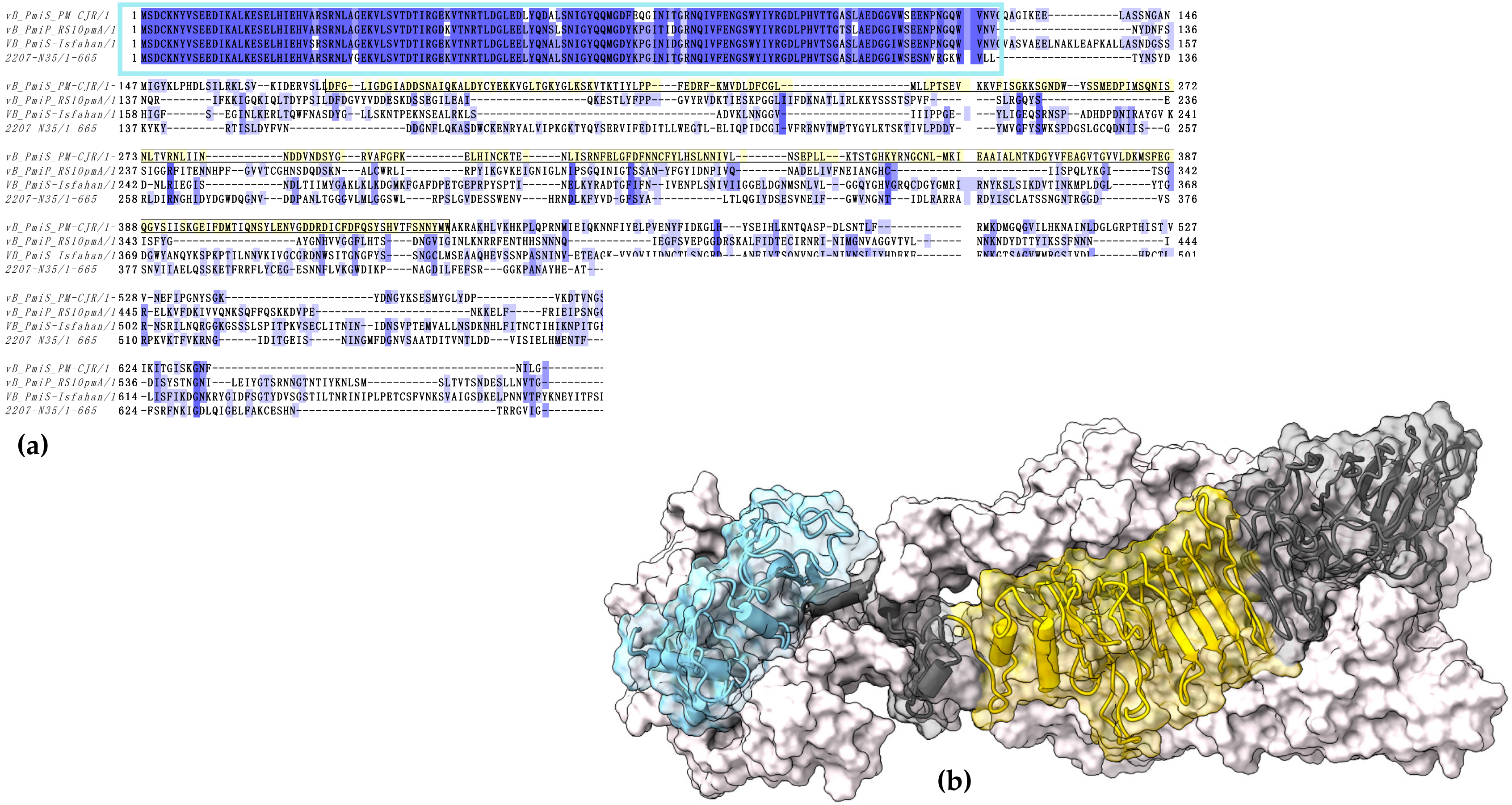

Figure S2. Bioinformatics analysis of the tail spike protein of vB_PmiS_PM-CJR. (**a**) Multiple sequence alignment of the tail spike protein sequences of vB_PmiS_PM-CJR and related *Gorganviruses*. The multiple alignment was constructed using MAFFT in L-INS-I mode and visualized with Jalview. Amino acid residues are colored by percentage identity. Conserved N-terminal region of tail spike proteins is indicated with a cyan box, amino acid residues of the catalytic pectin/pectate lyase fold domain (based on InterProScan prediction) are colored yellow. (**b**) Structural model of the vB_PmiS_PM-CJR tail spike based on an I-TASSER prediction. The trimeric model was assembled in UCSF ChimeraX. Conserved N-terminal region is shown in light blue, catalytic domain is shown in yellow.

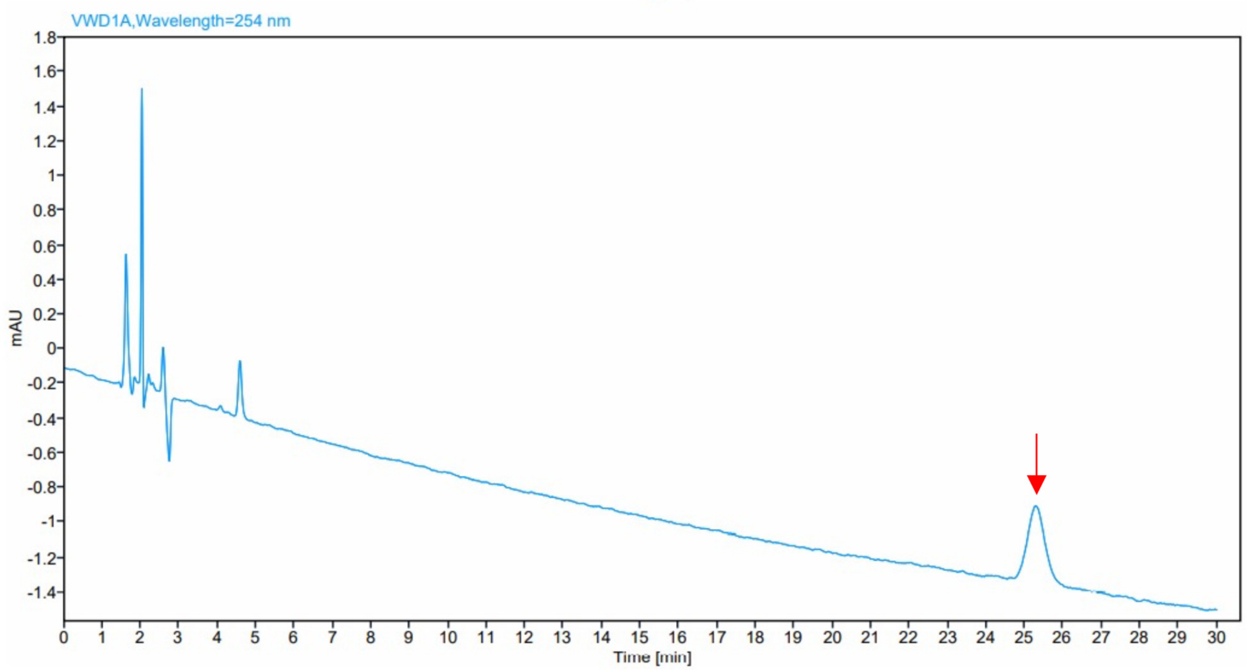

Figure S3. Purified tail spike purity analysis using reverse phase HPLC. A single peak (indicated with a red arrow) is visible at 25–26 min time mark.
